# Comparison of Single and Prolonged Fluoroquinolone Prophylaxis and Risk Factors for Infectious Complications After Transrectal Prostate Biopsy

**DOI:** 10.4274/balkanmedj.2018.0477

**Published:** 2018-09-21

**Authors:** Arif Kalkanlı, Cem Tuğrul Gezmiş, Arif Özkan, Nusret Can Çilesiz, Fatih Yanaral, Memduh Aydın, Zafer Tandoğdu

**Affiliations:** 1 Clinic of Urology, İstanbul Gaziosmanpaşa Taksim Training and Research Hospital, İstanbul, Turkey; 2 Clinic of Urology, İstanbul Haseki Training and Research Hospital, İstanbul, Turkey; 3 Department of Urology, Northern Institute for Cancer Research Newcastle University, Newcastle, United Kingdom

**Keywords:** Biopsy, fluoroquinolone, prophylaxis, prostate, urinary tract infections

## Abstract

**Background:**

The ideal prophylaxis duration for transrectal ultrasonography-guided prostate biopsy is incompletely defined.

**Aims:**

To compare the infectious complications of transrectal ultrasonography-guided prostate biopsy with and without extended antibiotic prophylaxis. The secondary aim was to evaluate the risk factors for infectious complications.

**Study Design:**

Prospective observational study.

**Methods:**

Four hundred patients who underwent transrectal ultrasonography-guided prostate biopsy were recruited. Patients orally received either 750 mg ciprofloxacin 60 min before the procedure or 500 mg ciprofloxacin twice a day for a duration of 7 days with the initial dose administered 24 h prior to the procedure. All patients were followed-up for 4 weeks after the transrectal ultrasonography-guided prostate biopsy procedure for infectious complications. Screening of urine was carried out in all patients on the 3^rd^ and 7^th^ day after the procedure. Medical histories of all patients were collected prior to biopsy. Information on medical history include the following: hospitalization, urethral catheterization, or urinary tract infections within the past 12 months; antibiotic use within the last 3 months, prior urinary tract interventions, and previous transrectal ultrasonography-guided prostate biopsy and Charlson comorbidity indexes. Ultrasound-guided biopsy was carried out using General Electric’s 7 MHz transrectal ultrasound device in the left decubitus position. Patients received one of the two ciprofloxacin-based prophylaxis regimens. Subsequent transrectal ultrasonography-guided prostate biopsy to all patients were followed-up for 30 days. Further follow-up of patients was carried out on the second and fourth weeks after transrectal ultrasonography-guided prostate biopsy, and symptoms, such as dysuria, rectal bleeding, fever, hematospermia, hematuria, and pollakiuria, were recorded.

**Results:**

Both groups presented similar baseline characteristics and medical history. Infectious complication rates within the 4-week follow-up were similar in both groups (single dose: 3% vs prolonged: 3%) (p>0.05). In both groups, infectious complications significantly increased than that at previous antibiotic usage (single: p=0.028; prolonged: p=0.040). Non-infectious complication ratios showed no significant variation (p>0.05).

**Conclusion:**

Pre-operative single dose of 750 mg oral ciprofloxacin compared with 7 days prolonged treatment resulted in similar infectious complication outcomes in patients undergoing transrectal ultrasonography-guided prostate biopsy. The use of antibiotics within the last 3 months increases the risk for post-transrectal ultrasonography-guided prostate biopsy infectious complications.

Within the last few decades, the prevalence of transrectal ultrasound-guided prostate biopsies (TRUS-bx) has increased worldwide owing to the use of prostate-specific antigen (PSA) for screening of prostate cancer (PCa) (1,2). Although much debate is still ongoing regarding the best practice approach on biopsy criteria, the increase in TRUS-bx has exposed a high number of men associated to its complications. A large proportion of these complications consists of minor complications, such as hematuria, dysuria, hematospermia, and rectal bleeding. However, the most dreaded complication is severe urinary tract infections (UTIs) such as sepsis (3). Antibiotic prophylaxis prior to TRUS-bx is administered to decrease the chances of infectious complications (4,5,6,7).

The increase in antimicrobial resistance (AMR) is a threat toward the success of TRUS-bx prophylaxis and to the whole health care system. An important reason for the increase in AMR is the high environmental antibiotic pressure. For this reason, antibiotic stewardship programs and controlled prophylaxis practice are important tools to use. Unfortunately, the misuse of antibiotics for prophylaxis commonly occurs in the field of urology (8). This situation is mainly due to the lack of evidence for best practice approaches to support decision making (8,9). For prophylaxis in TRUS-bx, fluoroquinolones are the most common preferred antibiotic group owing to the high concentrations that they can achieve in prostate tissues (3,6,5,7,8,9). Nevertheless, the duration at which these compounds should be administered for prophylaxis in TRUS-bx remains unclear, and prolonged prophylaxis regimens are common (8,9,10,11,12). This result conflicts with the definition of prophylaxis, which aims to avoid infections due to health care intervention in high-risk groups. Recent studies have shown no immediate additional benefit of prolonged antibiotic administration for prophylaxis; however, on the contrary, prolonged antibiotic administration is known to increase environmental antibiotic pressure (13,14). TRUS-bx-associated infectious complications can develop within the first 30 days of the intervention. To our knowledge, no study evaluated the 30-day infectious complication rates with extended-dose compared with single-dose prophylaxis. As an attempt to tailor prophylaxis, targeted approaches, for which related evidence remains unclear, have been developed (15,16,17,18). Prolonged duration of prophylaxis for TRUS-bx contributes to the already serious problem of AMR. Therefore, in this study, we aimed to identify if prolonged antibiotic administration for prophylaxis would benefit avoiding the 30-day infectious complications rates associated to TRUS-bx. In this study, we compared the infectious complication rates of ciprofloxacin-based single-dose prophylaxis against prolonged antibiotic administration as part of the prophylaxis for TRUS-bx.

## MATERIALS AND METHODS

### Design of the study

We conducted a prospective observational study in our hospital between February 2015 and December 2017. All patients who required a TRUS-bx for the suspicion of PCa were eligible for the study (Supplement 1). These patients received either a single-dose prophylaxis or prolonged antibiotic administration (details explained below). The exclusion criteria of the study are as follows:

- Previously known hypersensitivity to ciprofloxacin; 

- Gastrointestinal diseases preventing oral ciprofloxacin treatment;

- Diagnosis of UTI prior to TRUS-bx;

- History of endoscopic manipulation or catheterization within the last 10 days;

- Conditions that require particular antibiotic prophylaxis practices (e.g., endocarditis prophylaxis);

- Immunosuppression (AIDS, end-stage renal failure, and chronic steroid use).

**Figure 1:**
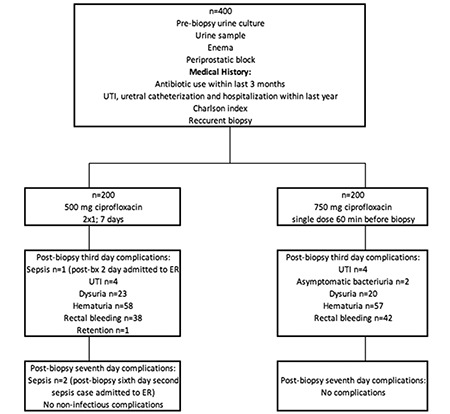
Design and survey for study.
*UTI: urinary tract infection*

Institutional ethical approval was received from the local ethical board. All patients were informed about the procedure, and possible complications and patient consenting for the study were included in the study. Overall, 437 patients agreed to join the study, and 400 completed the follow-ups. Medical histories of all the patients were collected prior to biopsy. The risk factors associated with the increased risk of health care-associated UTI were collected (19,20,21,22). These risk factors include the following: hospitalization, urethral catheterization, or UTI within in the past 12 months; antibiotic use within the last 3 months, prior urinary tract interventions, previous TRUS-bx, and Charlson comorbidity indexes. Patients with UTI were confirmed through urinalysis and urine culture test 3 days prior to biopsy. Patients with positive findings (pyuria and bacteriuria) were excluded (Figure 1).

### Biopsy procedure

Ultrasound-guided biopsy was carried out using General Electric’s 7 MHz transrectal ultrasound device. The biopsy was performed with patients in the left decubitus position and with their knees pulled up to their abdomen. Disposable needles were used for the procedure. Patients were asked not to use any anticoagulant or non-steroid anti-inflammatory medicine during the preceding week of the biopsy. Periprostatic local anesthetic was administered to all patients prior to the biopsy procedure (23). Following the European Association of Urology guidelines (23), a 12-core biopsy protocol was followed: two samples were obtained from each prostatic location (apex, mid, and basis of the prostate, both from the left and right sides). Additional cores were obtained from each transitional zone from patients who had previously undergone a prostate biopsy without findings of PCa (a total of 14 cores).

### Prophylaxis options

Patients received one of the two ciprofloxacin-based prophylaxis regimens. The first regimen included oral administration 500 mg ciprofloxacin twice a day for 7 days with the first dose started 24 h prior to the biopsy. The second regimen included a single oral dose of 750 mg ciprofloxacin 60 min before biopsy without any additional antibiotics.

### Patient follow-up

Subsequent to TRUS-bx, all patients were followed-up for 30 days. On the 3^rd^ and 7^th^ days, all patients were examined at the outpatient clinic and assessed for any symptoms of infection or complications related with the TRUS-bx. At these time points, urinalysis and urine culture were performed to capture asymptomatic urine findings. Further follow-up of patients was carried out on the second and fourth weeks after TRUS-bx, and symptoms, such as dysuria, rectal bleeding, fever, hematospermia, hematuria, and pollakiuria, were recorded. Any complications arising until the 30-day follow-up were recorded. These complications included hematuria, hematospermia, rectal bleeding, pain, dysuria, UTI, and retention. Diagnostic criteria of UTI and classification are provided in Supplement 2.

### Statistical analysis

Mean, standard deviation, median, lowest and highest scores, frequency, and ratio values were used for the descriptive statistics of the data. Distributions of variables were measured using Kolmogorov-Smirnov test. For the analyses of independent quantitative data, Mann-Whitney U test or chi square test was utilized; when these methods were inapplicable, Fischer’s exact test was used. All analyses were conducted with SPSS 22.0. The accepted level of statistical significance was p<0.05. The statistical power of our study was calculated post-hoc by free-for-public-use software: Post-hoc Power Calculator (web address: http://clincalc.com/stats/power.aspx). Using this software, the statistical power of the study was calculated to be 90.6%.

## RESULTS

### Demographics

Age (63.5±7.2 and 62.2±8.1. p=0.821), PSA levels (9.4±17.3 and 11.1±18.5. p=0.549), prostate volume (52.05±27.1 and 53.08±28.6. p=0.741), risk factors, Charlson score (2.34±1.3 and 2.15±1.1. p=0.477), and cancer detection rates (60 and 54; p=0.506) were similar for both groups (Table 1). PCa was detected in 114 (28.5%) patients and atypical small acinar proliferation (ASAP) in 20 (5%) patients. 

### Urine findings and clinical infectious complications

Follow-up urine culture screening with colony formation on the 3^rd^ [single: 2 (1%) vs prolonged: 2 (1%), p=0.001] and 7^th^ days [single: 3 (1.5%) vs prolonged: 2 (1%), p=0.001] post TRUS-bx were similar for both prophylaxis groups (Table 2). Symptomatic UTI within the 30-day follow-up period was identified in 8 patients (2%) (single: 4, prolonged: 4, p=0.001) from both groups. In single-dose prophylaxis group, 5 patients showed growth in urine culture. Two patients indicated asymptomatic bacteriuria (ABU). Three patients who received single-dose prophylaxis and developed UTI with growth in urine culture and follow-up were managed with antibiotics at the outpatient setting and required no additional interventions. The last patient with infectious complication in the single-dose prophylaxis group showed no growth in urine culture but presented pyuria, dysuria, and suprapubic tenderness. In the prolonged administration group, two patients who developed UTI with microbiological findings developed severe infections [systemic inflammatory response syndrome (SIRS) and sepsis] and required hospitalization and additional treatments. Two patients in the prolonged administration group and who developed UTI exhibited no growth in the urine culture but manifested pyuria, dysuria, and suprapubic pain. The last two patients who developed UTI in the prolonged administration group showed growth in urine culture (Table 2).

### Risk factors for infectious complications

Table 3 summarizes the results of subgroup analysis of patients in the prophylaxis groups according to the measured risk factors. The distributions of these risk factors differed in both groups. Further univariate analysis  of factors impact on symptomatic UTI was identified and previous antibiotic usage within 3 months was significantly increases risk of symptomatic UTI for both groups (single: p=0.0289; prolonged: p=0.0405). The effects of other risk factors showed no significant variation (p>0.05) (Table 3).

### Non-infectious complications

The most common non-infectious findings comprised microscopic hematuria [single: 57 (28.5%) vs prolonged: 58 (29%) p=0.001] followed by rectal bleeding [single: 42 (21%) vs prolonged: 38 (19%) p=0.001] and dysuria [single: 20 (10%) vs prolonged: 23 (11.5%) p=0.001]. Non-infectious complication rates were similar in both groups (p<0.05) (Table 4). One patient from the long-term treatment group developed urinary retention, for which urethral catheterization was performed. Following TRUS-bx, hospitalization was deemed unnecessary for any condition other than infection.

## DISCUSSION

Our study has identified no additional benefit of extending the antibiotic administration in patients who undergo TRUS-bx. Urology guidelines strongly recommend antimicrobial prophylaxis. However, the choice of regimens and duration of prophylaxis remains debatable (23). Furthermore, the literature lacks both the definition for the risk factors and the benefits of prolonged antibiotic administration in this specific group. As part of this ongoing uncertainty, the current practice of prophylaxis in TRUS-bx is heterogeneous, leading to increased antibiotic usage (8,9). Annually, over a million prostate biopsies are performed in Europe. Despite the emerging new approaches to perform biopsies with multiparametric magnetic resonance imaging, overall, TRUS-bx currently remains a common practice. The misuse of antibiotic for prophylactic purposes is well known (24,25) and is mostly applied to avoid complications, such as severe infections requiring hospitalization. In our study, 2% of cases had to be hospitalized due to infections despite prolonged antibiotic administration. A similar trend has also been reported by other studies (3,26). These findings represent the failure of antibiotic prophylaxis and are a direct consequence of the increase in AMR. The prevalence of AMR depends on a complex network, which includes local antibiotic pressure, infection control policies, veterinary antibiotic usage, and sanitation. Therefore, antibiotic options should be tailored for each environment. In our study environment, the AMR rates are one of the highest in the world (8,19). Thus, prior to starting the study, we audited our annual AMR rates for infections in single-day outpatient urological interventions. Fluoroquinolones, the first line recommended antibiotic for prophylaxis in TRUS-bx, AMR rate was below 20%. Hence, in our study, we proceeded with the ciprofloxacin regimens to be tested. Similar rates of UTI after TRUS-bx with fluoroquinolone-based antibiotic regimen prophylaxis were identified in other parts of the world (14). The reported rates of infectious complications following TRUS-bx range from 0.1% to 20% (27). In our study, among the 400 patients receiving quinolone prophylaxis, 2 (0.25%) developed fever, and 12 (3%) presented total infectious complications (ABU, UTI, and sepsis). Nevertheless, in our study, the infection rates were relatively high. Therefore, the use of fluoroquinolones is questionable in our region. Many recently conducted studies indicate a progressive increase in the ciprofloxacin-resistant ESBL (+) bacilli rates in pre-biopsy fecal cultures (16,17,18,28). In this study, quinolone-resistant bacteria grew in the cultures of all patients who have developed infectious complications. Patients with this type of bacteria in their intestinal flora feature higher rates of infection after TRUS-bx (15). This result suggests a need for new prophylaxis regimens (15,16,17,18). Collecting rectal culture prior to TRUS-bx may be an option to overcome this problem, but its application for each patient may cause trouble for urologists. Steensels et al. (16) suggest rectal cultures only for TRUS-bx patients who meet the risk factors for infections, such as quinolone use in the last six months, recurrent UTI or prostatitis, and history of infectious complication in previous TRUS-bx. Another main finding in our study was the high rate of antibiotic use in our country compared with that reported in the literature. A total of 112 of the 400 patients had used antibiotics in the last 3 months; this number, at a 28% rate, is higher than the 15% reported by other studies. This result was related with the practice of antibiotics use in Turkey in high-PSA-level patients in 76 of the 112 patients using previous antibiotics in our study. The study has been conducted in a prospective observational manner and cannot control for selection bias. In our study, the prophylaxis regimen selection was carried out by the urologist responsible for the TRUS-bx. Unless any particular reason (i.e., endocarditis prophylaxis and allergies) was provided, the prophylaxis regimen was selected among the two options for all patients participating in the study. Despite the non-randomized nature of the study, the two prophylaxis arms featured similar measurable baseline characteristics. As a further attempt to minimize the selection bias, the study was implemented as a departmental practice protocol for its whole duration. All patients attending the department for TRUS-bx were approached for the study by a member of the research team. We identified that 90.45% of patients who were approached accepted to join the study. Despite all these attempts to minimize the effect of selection bias, we suggest that the results should be approached with caution. The prevalence of serious UTIs following TRUS-bx can range between 0.1% to 4% (3.26). Therefore, capturing the true effect of prolonged duration antibiotic administration for prophylaxis would require a much larger sample size. However, our current results show no support on the use of prolonged antibiotic administration for prophylaxis. Our study suggests that single-dose 750 mg oral ciprofloxacin provides similar infection rates when compared with 7 days of prolonged treatment in patients undergoing TRUS-bx. Furthermore, our results indicate that use of antibiotics within the last 3 months increases the risk for post-TRUS-bx infectious complications. These findings require validation with larger studies and also should prompt surveillance of departmental practices to obtain locally relevant data.

## Figures and Tables

**Table 1:**
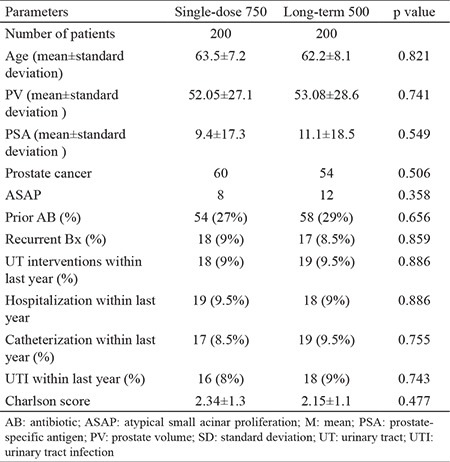
General patient characteristics

**Table 2:**
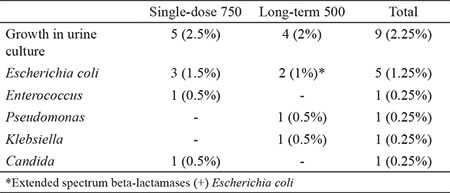
Significant growth findings in the urine culture following TRUS-bx

**Table 3:**
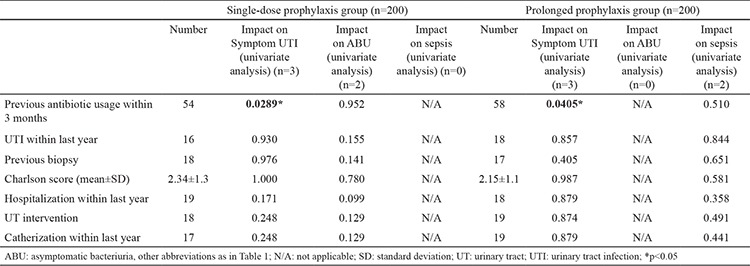
Effects of risk factors on infectious complications

**Table 4:**
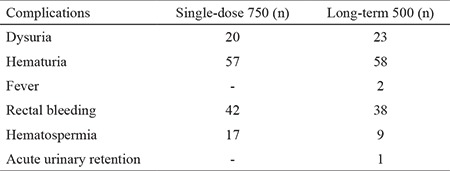
Post-operative complications
